# Revisiting chromatin binding of the Arabidopsis UV-B photoreceptor UVR8

**DOI:** 10.1186/s12870-016-0732-5

**Published:** 2016-02-11

**Authors:** Melanie Binkert, Carlos D. Crocco, Babatunde Ekundayo, Kelvin Lau, Sarah Raffelberg, Kimberley Tilbrook, Ruohe Yin, Richard Chappuis, Thomas Schalch, Roman Ulm

**Affiliations:** Department of Botany and Plant Biology, University of Geneva, Sciences III, 30 Quai E. Ansermet, CH-1211 Geneva 4, Switzerland; Department of Molecular Biology, University of Geneva, Sciences III, 30 Quai E. Ansermet, CH-1211 Geneva 4, Switzerland; Institute of Genetics and Genomics of Geneva (iGE3), University of Geneva, CH-1211 Geneva 4, Switzerland; Present Address: CSIRO Agriculture, Canberra, Australia

**Keywords:** *Arabidopsis*, ChIP, UV-B, UVR8, Chromatin, RCC1, Photoreceptor

## Abstract

**Background:**

Plants perceive UV-B through the UV RESISTANCE LOCUS 8 (UVR8) photoreceptor and UVR8 activation leads to changes in gene expression such as those associated with UV-B acclimation and stress tolerance. Albeit functionally unrelated, UVR8 shows some homology with RCC1 (Regulator of Chromatin Condensation 1) proteins from non-plant organisms at the sequence level. These proteins act as guanine nucleotide exchange factors for Ran GTPases and bind chromatin via histones. Subsequent to the revelation of this sequence homology, evidence was presented showing that UVR8 activity involves interaction with chromatin at the loci of some target genes through histone binding. This suggested a UVR8 mode-of-action intimately and directly linked with gene transcription. However, several aspects of UVR8 chromatin association remained undefined, namely the impact of UV-B on the process and how UVR8 chromatin association related to the transcription factor ELONGATED HYPOCOTYL 5 (HY5), which is important for UV-B signalling and has overlapping chromatin targets. Therefore, we have investigated UVR8 chromatin association in further detail.

**Results:**

Unlike the claims of previous studies, our chromatin immunoprecipitation (ChIP) experiments do not confirm UVR8 chromatin association. In contrast to human RCC1, recombinant UVR8 also does not bind nucleosomes in vitro. Moreover, fusion of a VP16 activation domain to UVR8 did not alter expression of proposed UVR8 target genes in transient gene expression assays. Finally, comparison of the Drosophila DmRCC1 and the Arabidopsis UVR8 crystal structures revealed that critical histone- and DNA-interaction residues apparent in DmRCC1 are not conserved in UVR8.

**Conclusion:**

This has led us to conclude that the cellular activity of UVR8 likely does not involve its specific binding to chromatin at target genes.

**Electronic supplementary material:**

The online version of this article (doi:10.1186/s12870-016-0732-5) contains supplementary material, which is available to authorized users.

## Background

Photosynthetic plant life is exposed to potentially damaging UV-B radiation (280–315 nm; herein UV-B) which is intrinsic to sunlight. UV-B photons are specifically and sensitively detected in plants by the UV-B photoreceptor UV RESISTANCE LOCUS 8 (UVR8) [1–5]. UVR8-mediated signalling is known to culminate in altered expression of a broad range of genes [6, 7] which ultimately leads to UV-B acclimation and UV-B tolerance. UVR8 UV-B signalling involves the interaction of UVR8 with the E3 ubiquitin ligase CONSTITUTIVELY PHOTOMORPHOGENIC 1 (COP1) and associated stabilization of the bZIP transcription factor ELONGATED HYPOCOTYL 5 (HY5) [7–9]. HY5 then influences expression of a number of UV-B regulated genes [10], including expression of *HY5* itself [11]. Indeed, a T/G-box *cis*-regulatory element within the *HY5* promoter functions as an HY5 binding site and is required for UV-B-induced *HY5* expression [11, 12].

Apart from HY5 stabilization, past reports have proposed a more direct mechanism of UVR8 regulation of UV-B-dependent transcription. UVR8 has sequence similarity to the eukaryotic guanine nucleotide exchange factor Regulator of Chromatin Condensation (RCC1), which interacts with chromatin and specifically with histones [13]. RCC1 proteins act as guanine nucleotide exchange factors (GEF) and regulate the small GTPase Ran. RCC1 is essential to critical cellular processes in eukaryotes such as nucleocytoplasmic transport, nuclear envelope formation, and spindle assembly during mitosis [14–17]. Although UVR8 is not otherwise functionally related to RCC1, chromatin immunoprecipitation (ChIP) assays have suggested that UVR8 binds chromatin in vivo [6, 18–20]. It is tempting to assume that UVR8 chromatin association is due to structural similarity with RCC1 proteins. Recent studies of RCC1 structure illustrate how this protein interacts with histone and DNA components of the nucleosome [21, 22]. RCC1 is a β-propeller protein that interacts with histones and nucleosomal DNA through a ‘switchback loop’ region and its N-terminal tail [22]. RCC1 and histones make contact through the H2A-H2B histone dimer surface of the nucleosome core particle whilst contacts between RCC1 and DNA are made through the DNA phosphate backbone. This indicates that RCC1 interacts with chromatin by binding non-DNA-sequence specific areas [21]. Consistently, the yeast RCC1-orthologue Srm1/Prp20 was found to bind most nucleosomes in the genome with no apparent sequence specificity [23].

Similar unspecific binding was indicated for Arabidopsis UVR8 by reports of its association with a region larger than 3 kb around the *HY5* genomic locus and its chromatin association via histones, preferentially histone H2B [6, 19]. However, in contrast to the yeast RCC1, the proposed UVR8 chromatin association was confined to specific genes. Of the promoter regions tested, UVR8 was found to interact with chromatin of some (e.g., At5g11260, *HY5*; At5g24850, *CRY3*; At2g47460, *PFG1*/*MYB12*) but not all (e.g., At3g17609, *HYH*; At5g13930, *CHS*) UVR8-regulated genes [19]. However, both the specificity of UVR8 for particular target genes and the mechanism conferring specificity need to be more firmly established.

Association of Arabidopsis UVR8 with chromatin of UVR8 target genes was reported to be constitutive and not UV-B-responsive [6, 19], despite the fact that UVR8 accumulates in the nucleus upon UV-B irradiation [18]. Thus, independent of the ambiguities surrounding target gene specificity, it is also a valid question whether UVR8 chromatin association indeed underlies UVR8 involvement in transcription regulation and, if so, how UVR8 influences gene expression mechanistically. It has been suggested that UVR8 is involved in the recruitment of transcriptional regulators and/or in chromatin remodelling [19].

Here, we have experimentally revisited chromatin association of UVR8 and suggest that a mode-of-action involving direct UVR8 chromatin association should be considered with caution and alternative mechanisms should not be dismissed.

## Results and discussion

### Arabidopsis UVR8 does not associate with promoter regions of *HY5* and *MYB12* target genes in quantitative ChIP qRT-PCR assays

**Fig. 1:**
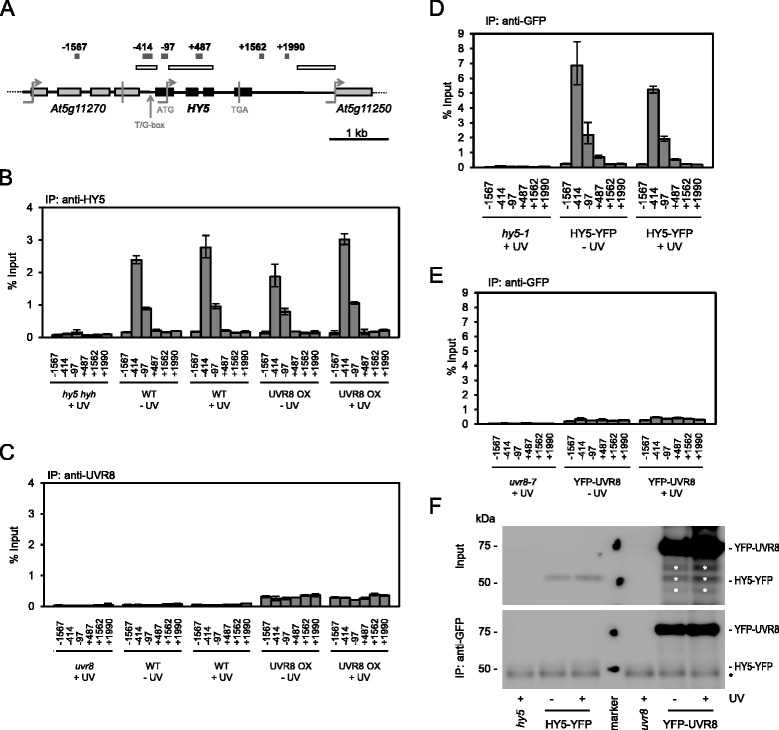
UVR8 does not associate with the *HY5* genomic region. **a** Schematic representation of the *HY5* genomic locus and surrounding region. *Black* and *grey boxes* depict the *HY5* transcribed region (with start ATG and stop TGA of *HY5* indicated) and portions of the transcribed regions of the neighbouring genes, respectively. *Upper grey bars* indicate qRT-PCR amplicons at various positions at and surrounding the *HY5* genomic locus with numbers indicating the position of the 5’ end of the amplicon relative to the *HY5* translation start site (referred to as position +1). An *arrow* indicates the position of the T/G-box *cis*-element bound by HY5 [11, 12]. Open bars are placed at genomic regions previously reported to be UVR8 binding targets in gel-based ChIP assays [19]. **b**, **c** ChIP-qPCR of DNA associated with HY5 (**b**) and UVR8 (**c**) presented as the percentage of total input DNA recovered by immunoprecipitation (% Input). The same chromatin pool was used for immunoprecipitation with both HY5 and UVR8 antibodies. WT = Ws/*Pro*_*HY5*_ *:Luc+*, UVR8 OX = Ws/*Pro*_*HY5*_ *:Luc + Pro*_*35S*_ *:UVR8. Arabidopsis* mutant lines with corresponding genetic backgrounds (*uvr8* = *uvr8-7*/*Pro*_*HY5*_ *:Luc+*, *hy5 hyh* = *hy5-ks50 hyh-1*/*Pro*_*HY5*_ *:Luc+*) were included as negative controls. **d**, **e** As in (**b**, **c**), but of DNA associated with HY5-YFP (**d**) and YFP-UVR8 (**e**), performed using the same anti-GFP antibody for immunoprecipitation. HY5-YFP = *hy5-1*/*Pro*_*35S*_ *:HY5-YFP*, YFP-UVR8 = *uvr8-7*/*Pro*_*35S*_ *:YFP-UVR8. Arabidopsis* mutant lines with corresponding genetic backgrounds (*hy5-1* and *uvr8-7*) were included as negative controls. **b**, **c**, **d**, **e** The number labelling indicates the location of each analysed DNA fragment, specifically the position of the 5’ end of the amplicon relative to the translation start site (referred to as position +1). Experimental material was sourced from ten-day-old seedlings, either processed with no UV-B exposure (−UV) or following 4 h UV-B (+UV). Error bars represent SDs of three technical replicates. **f** Input and eluate of D/E ChIP samples were analysed on western blots with an antibody against GFP. Bands resulting from degradation products (upper panel) and unspecific background signal (*lower panel*) are marked (*). Molecular weight marker positions representing 50 and 75 kDa are shown (*marker lane*)

To better understand the dynamics and role of the proposed UVR8 chromatin association, we set out to establish whether UVR8 physically interacted with chromatin at previously described target genes using quantitative ChIP qRT-PCR. We first tested association of endogenous UVR8 with the *HY5* genomic locus using several target regions for which positive qualitative gel-based ChIP has been described [19] (Fig. 1a). Our anti-UVR8 antibody was raised against the same peptide (representing amino acids 410–424) used in previous ChIP experiments [19]. This antibody is highly efficient and specific both in western blotting and co-immunoprecipitation of UVR8 with endogenous COP1 [24, 25]. To ensure adequate crosslinking conditions and a functional ChIP protocol, we used the same cross-linked chromatin pool to perform ChIP of HY5 using an anti-HY5 antibody and processed the samples in parallel to those arising from UVR8 ChIP.

HY5 ChIP clearly confirmed binding of HY5 to its own promoter (Fig. 1b and Additional file 1a), as has been previously documented [11, 12]. However, in contrast to previous reports [6, 18–20], we did not detect UVR8 chromatin association with *HY5* using the anti-UVR8^(410–424)^ antibody in conjuction with several different probes covering a large portion of the *HY5* genomic region (Fig. 1c and Additional file 1a), including those regions previously reported to be bound by UVR8. Similar to wild type, we also did not detect UVR8 chromatin association with *HY5* in a transgenic line overexpressing UVR8 (UVR8 OX, Fig. 1c).

To ensure comparable antibody functionality during immunoprecipitation, we performed further ChIP qRT-PCR assays and used the exact same antibody for both HY5 and UVR8. For this, we resorted to the *Pro*_*35S*_*:YFP-UVR8*, *Pro*_*35S*_*:CFP-UVR8* and *Pro*_*35S*_*:HY5-YFP* transgenic lines and used a polyclonal GFP (green fluorescent protein) antibody to perform ChIPs of HY5-YFP and YFP-UVR8 or CFP-UVR8 in parallel (note that YFP and CFP differ from GFP in only a few amino acids and are, thus, efficiently recognized by anti-GFP antibodies). In agreement with our HY5 ChIP data, we obtained a clear signal for HY5-YFP binding to the endogenous *HY5* promoter (Fig. 1d and Additional file 1b). However, as for UVR8 ChIP, we did not detect any specific *HY5* signal above background in YFP-UVR8 and CFP-UVR8 ChIP assays, a result that was mirrored in the non-transgenic negative controls (*hy5-1*, *uvr8-7*, Col WT) (Fig. 1e and Additional file 1c).

To verify that YFP-UVR8, CFP-UVR8 and HY5-YFP were efficiently immunoprecipitated with the anti-GFP antibody during the ChIP procedure, we analysed the protein-chromatin eluate by western blotting. Following the immunoprecipitation step, we found YFP-UVR8 and CFP-UVR8 in the eluate and at positions in agreement with their expected size of 74 kDa (47 kDa UVR8 plus 27 kDa YFP/CFP) (Fig. 1f and Additional file 1d). HY5-YFP was detectable in the input and IP fractions (Fig. 1f and Additional file 1d) although it ran slightly higher than its predicted molecular weight of 45 kDa (18 kDa HY5 plus 27 kDa YFP). However, a similar size discrepancy has been observed previously (endogenous HY5 migrating at 30 kDa) [26]. It is of note that tagged UVR8 proteins were expressed and immunoprecipitated at higher levels than HY5-YFP, and yet, unlike HY5-YFP, YFP-UVR8 and CFP-UVR8 ChIP assays did not result in *HY5* ChIP signal (Fig. 1 and Additional file 1).

**Fig. 2:**
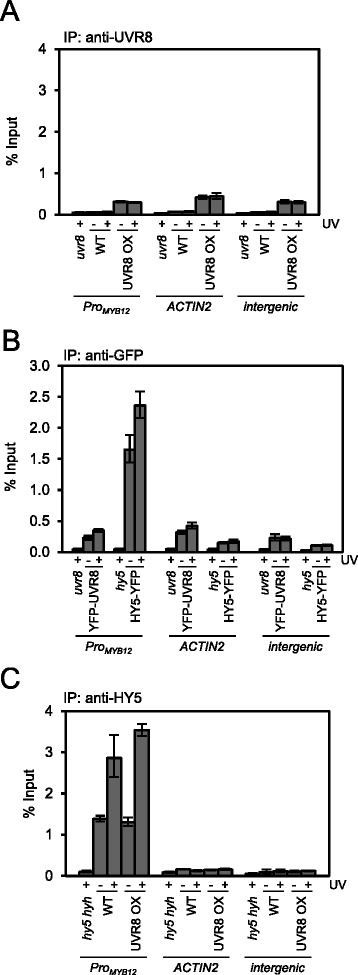
UVR8 does not bind to the *MYB12* promoter. **a** UVR8 chromatin association in wild-type plants (WT = Ws/*Pro*_*HY5*_ *:Luc+*), a *uvr8* mutant (*uvr8-7*/*Pro*_*HY5*_ *:Luc+*) and a UVR8-overexpression line (UVR8 OX = Ws/*Pro*_*HY5*_ *:Luc + Pro*_*35S*_ *:UVR8*). ChIP-qPCR was performed using an anti-UVR8^(410–424)^ antibody for immunoprecipitation. **b** Chromatin association in HY5-YFP expressing plants (*hy5-1*/*Pro*_*35S*_ *:HY5-YFP*), a YFP-UVR8 line (*uvr8-7*/*Pro*_*35S*_ *:YFP-UVR8*) and *hy5-1* and *uvr8-7* mutant controls. ChIP-qPCR was performed using an anti-GFP antibody for immunoprecipitation. **c** HY5 chromatin association in wild-type plants (WT = Ws/*Pro*_*HY5*_ *:Luc+*), a UVR8 overexpression line (UVR8 OX = Ws/*Pro*_*HY5*_ *:Luc + Pro*_*35S*_ *:UVR8*) and *hy5 hyh* mutant control (*hy5-ks50 hyh-1*/*Pro*_*HY5*_ *:Luc+*). ChIP-qPCR was performed using an anti-HY5 antibody. **a**, **b**, **c** Chromatin association was analysed by ChIP-qPCR for the *MYB12* promoter (*Pro*_*MYB12*_), *ACTIN2* and an intergenic control region between the *At4g26900* and *At4g26910* genes. The analysed chromatin was the same as in the experiments presented in Fig. 1. Error bars represent SDs of three technical replicates

We further tested UVR8 binding to the promoter of *MYB12*, which is also a reported target gene of UVR8 [19] and compared genomic association to that of HY5 [11, 27, 28]. The primer pair used to detect the *MYB12* promoter amplifies a region that is included in the region amplified with a primer pair used previously [19]. However, as for the *HY5* target gene, we did not detect binding of UVR8, YFP-UVR8 or CFP-UVR8 to the *MYB12* promoter region above background ChIP signal for a non-UV-B regulated gene (*ACTIN2*) and a control intergenic region (Fig. 2a and b and Additional file 1e). In contrast, clear *MYB12* ChIP signal resulted from both ChIP of HY5, performed in parallel using an anti-HY5 antibody on the same cross-linked wild-type chromatin pool (Fig. 2c), and ChIP of HY5-YFP, performed with the same GFP antibody in transgenic lines (Fig. 2b and Additional file 1e). Indeed, UV-B enhanced the association of HY5 with the *MYB12* promoter region (Fig. 2b and c and Additional file 1e), as described before [11].

Taken together, the results of our quantitative ChIP qRT-PCR assays of endogenous UVR8 and transgenically expressed YFP-UVR8 and CFP-UVR8 do not support an association of UVR8 with previously reported chromatin regions. However, it cannot be excluded that differences in plant growth conditions or ChIP procedure may explain the discrepancy between our and previously published results. Independent of this, a functional role of UVR8 at the chromatin level needs to be reconsidered.

### VP16-UVR8 does not enhance expression of *HY5* and *MYB12* in transient protoplast assays

**Fig. 3:**
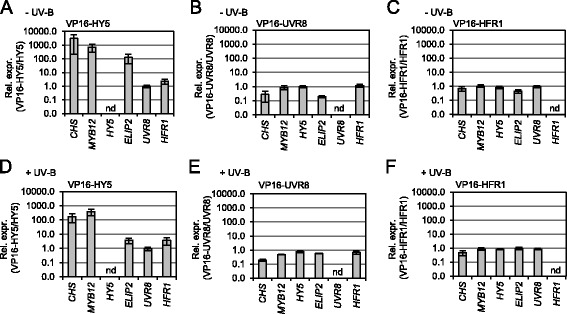
Fusion of the VP16 activator domain to UVR8 does not affect expression of suggested target genes. **a**, **b**, **c**, **d**, **e**, **f** The effect of fusing the VP16 activator domain to HY5 (VP16-HY5: **a**, **d**), UVR8 (VP16-UVR8: **b**, **e**) and HFR1 (VP16-HFR1: **c**, **f**) as measured by expression changes of target and negative control genes. Mutant protoplasts prepared from *hy5-ks50*, *uvr8-7* and *hfr1-101* seedlings were transfected with *Pro*_*35S*_ *:3xHA-VP16AD-HY5*/*Pro*_*35S*_ *:HY5*, *Pro*_*35S*_ *:3xHA-VP16AD-UVR8*/*Pro*_*35S*_ *:UVR8* and *Pro*_*35S*_ *:3xHA-VP16AD-HFR1*/*Pro*_*35S*_ *:HFR1* respectively. Gene expression was analysed 19–24 h post-transfection with protoplasts maintained under weak white light (**a**, **b**, **c**) and weak white light plus a final 3 h UV-B irradiation (**d**, **e**, **f**). Gene expression levels were normalized with the expression levels of the respective transfected effectors (i.e., *VP16-HY5*, *HY5*, *VP16-UVR8*, *UVR8*, *VP16-HFR1*, *HFR1*). Data is presented as relative expression in the presence of the effectors fused to VP16 to the respective control effectors without the VP16 domain (VP16-effector/effector). nd = non-determinable. Data are shown as the means of four (**a**, **b**, **c**) or three (**d**, **e**, **f**) biological replicates ± standard errors

As an alternative test of promoter association, we generated synthetic transcriptional activators by creating fusion proteins with the Herpes Simplex Virus VP16 acidic activation domain [29] and analysed the effect on target gene expression in a transient protoplast assay. To have a comparative means to guage the effect of UVR8 (VP16-UVR8), we used the established DNA-binding protein HY5 [30] as a positive control (VP16-HY5) and the non-DNA-binding HFR1 [31] as a negative control (VP16-HFR1). Transient expression of VP16-HY5 in Arabidopsis protoplasts strongly enhanced expression of its target genes (*CHS, MYB12, ELIP2*) but not of others (*UVR8, HFR1*) (Fig. 3a). This is in agreement with previous experiments showing strong activation of *CHS* and *MYB12* in stable transgenic Arabidopsis lines expressing VP16-HY5 fusion proteins [27]. In contrast, VP16-UVR8 did not activate the expression of the suggested direct UVR8 target genes (*MYB12, HY5*), either in white light (Fig. 3b) or in response to UV-B (Fig. 3e). It is, however, noteworthy that UVR8 can act as a UV-B photoreceptor in Arabidopsis protoplasts, as was evident by expression induction of marker genes *HY5* and *MYB12* in response to UV-B in protoplasts derived from wild-type seedlings but not from *uvr8-7* null mutants (Additional file 2). Moreover, in *uvr8-7*-derived protoplasts, transient expression of wild-type UVR8 was seen to complement the mutant phenotype (Additional file 2). In agreement with protoplast UV-B-responsiveness, enhanced target gene expression associated with VP16-HY5 was reduced when protoplasts were treated with UV-B (compare Fig. 3d with Fig. 3a), reflecting the enhanced basal expression levels of the *ELIP2, CHS* and *MYB12* target genes resulting from a typical transcriptional response to UV-B. Conversely, VP16-HFR1 expression had no effect on *CHS*, *MYB12*, *ELIP2*, *HY5* or *UVR8* mRNA levels (Fig. 3c and f), in agreement with the absence of DNA-binding activity of HFR1 [31].

### Drosophila RCC1 chromatin and DNA binding residues are not conserved in Arabidopsis UVR8

**Fig. 4:**
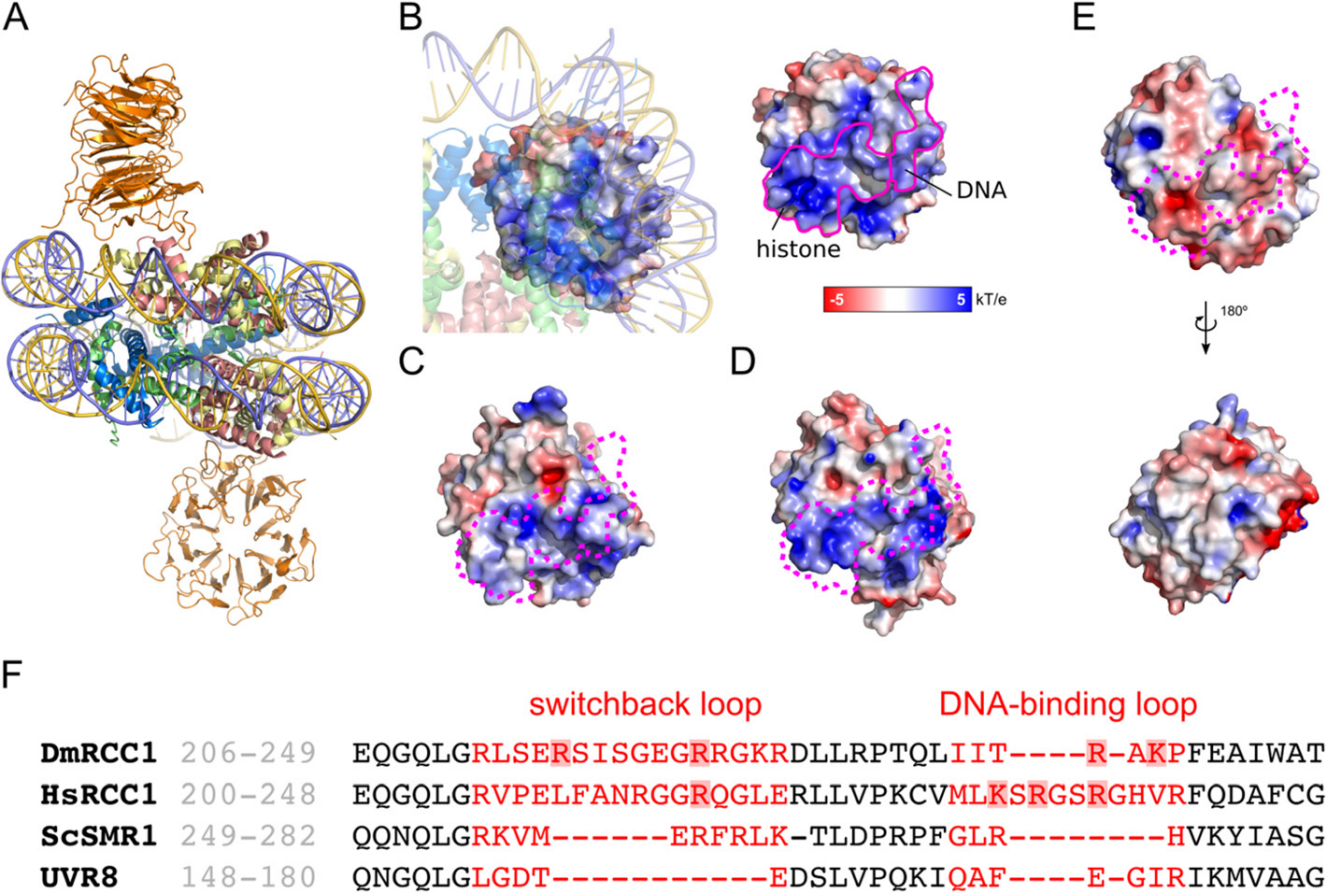
Structural comparison of UVR8 with the nucleosome binding interface of RCC1 shows that UVR8 lacks positive surfaces to interact with nucleosomes. **a** Overview of the Drosophila RCC1-nucleosome complex determined by X-ray crystallography [21]. Both RCC1 molecules bind identically to the nucleosome. **b** DmRCC1 viewed through the nucleosome shows the nucleosome interaction interface. Left: For RCC1, the solvent accessible surface is shown coloured in terms of electrostatic potential [52, 53]. The nucleosome is depicted as semi-transparent. Right: The same representation of RCC1 with the nucleosome contact surface outlined in pink. DNA and histone-binding regions are indicated. **c**, **d**, **e** Comparison of electrostatic surface potential of HsRCC1 (pdb 1A12) (**c**), ScSMR1/Prp20 (pdb 3OF7) (**d**) and UVR8 (pdb 4DNW) (**e**, upper) with an orientation identical to that of DmRCC1 in (**b**). The electrostatic surface potential for the opposite side of UVR8 is also shown by rotation of the molecule (**e**, lower). PyMOL (http://www.pymol.org) and APBS plugin for PyMOL (MG Lerner and HA Carlson, 2006, University of Michigan, Ann Arbor) was used for visualization of these structures. **f** Structure-based multiple sequence alignment of RCC1 homologs with UVR8. Shaded residues indicate those confirmed or proposed to be critical for RCC1 binding to the nucleosome

The initial investigation of UVR8 chromatin association was inspired by the sequence similarity of UVR8 with eukaryotic RCC1 proteins [6, 13], prior to publication of the UVR8 crystal structure [32, 33]. RCC1 binds chromatin as part of its biological activity in establishing a RanGTP/RanGDP gradient, with an increasing ratio in decreasing distance to chromatin [14, 15, 34]. The crystal structure of *Drosophila melanogaster* RCC1 (DmRCC1) in association with a nucleosome core particle (Fig. 4a) revealed two distinct binding sites, a histone- and a DNA-binding loop [21]. The DmRCC1 histone-binding loop is made up of two key arginine residues (Arg-216 and Arg-223) supported by a serine residue (Ser-214) that mediates the interaction with an acidic histone patch [21]. On the other hand, the DNA-binding activity of DmRCC1 is mediated by Gln-259, Lys-241 and Arg-239 through sequence-unspecific interaction with the DNA phosphate backbone [21]. Although crystal structures of *Saccharomyces cerevisiae* (ScSMR1/Prp20p) and human (HsRCC1) RCC1 proteins have been described and both proteins shown to bind nucleosomes, the molecular details of the chromatin interaction are less well understood [23, 34–38]. Structure comparison showed that critical arginine residues of the histone-binding loop are conserved, Arg-260 in ScSMR1/Prp20p and Arg-217 in HsRCC1, corresponding to DmRCC1 Arg-223 [35]. Furthermore, HsRCC1 Arg-217 was shown experimentally to be necessary for nucleosome interaction [22] and the mutation of basic residues in the DNA-binding loop of HsRCC1 showed Lys-232, Arg-234 and Arg-237 to be important for DNA binding [21].

The recent publication of the crystal structure of Arabidopsis UVR8 [32, 33] allowed us to carry out a structural comparison of Arabidopsis UVR8 with DmRCC1 to check for the presence in UVR8 of a conserved nucleosome-binding surface that is critical for histone and DNA interaction in DmRCC1. In addition, we compared DmRCC1 and ScSMR1/Prp20p as well as DmRCC1 and HsRCC1 (Fig. 4). As the free structures of HsRCC1, ScSMR1/PrP20p, and UVR8 are likely to undergo structural rearrangements in the switch-back loop and the DNA-binding surface upon binding to nucleosomes, it is difficult to compare individual side-chain positions. Thus, we chose to compare the electrostatic surface potentials as a measure of histone and DNA interaction potential. Since both DNA and the negative patch on the nucleosome surface are negatively charged, DmRCC1 shows strong accumulation of positive charge on both the histone- and DNA-interacting surfaces (Fig. 4b). Aligning the structures of HsRCC1 and ScSMR1/PrP20 revealed similar positive surface patches (Fig. 4c and d). However, UVR8 showed a rather negatively charged surface across the entire predicted nucleosome interaction surface, which argues strongly against an RCC1-like binding mode to nucleosomes. In addition, the UVR8 surface is generally devoid of strongly positively charged patches (Fig. 4e, upper and lower) and, thus, does not display the characteristics of a bona fide DNA-binding protein. Comparing structure-based sequence alignments, it became evident that the overall structural similarity between the UVR8 and DmRCC1 7-bladed β-propeller structures is striking but the histone-binding loop in DmRCC1 is not conserved in UVR8 (Fig. 4f). Similarly, we did not find a potential DNA-binding loop in UVR8 through structural comparison with DmRCC1. We conclude that the presence of histone- and DNA-binding surfaces is a general feature of the chromatin association of eukaryotic RCC1 proteins but these are apparently absent in Arabidopsis UVR8. This suggests that UVR8 may have lost the ability to bind to chromatin, corresponding to the differences in activity between the UVR8 and RCC1 proteins.

**Fig. 5:**
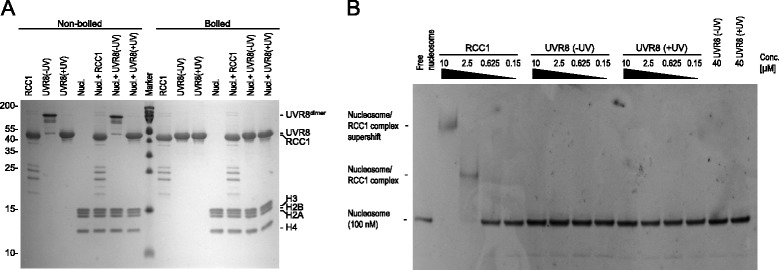
UVR8 does not bind to nucleosomes in vitro. **a** Purified recombinant RCC1 and UVR8 (− and + UV-B treatment) were electrophoretically separated on SDS-PAGE together or in the absence of reconstituted nucleosomes (Nucl.) and stained by Coomassie Blue. Samples were boiled or non-boiled before loading the gel. **b** Electrop horetic mobility gel shift assay of nucleosome binding by RCC1 and UVR8. Between 0.15 - 10 μM RCC1 and 0.15 - 40 μM UVR8 (− and + UV-B treatment) were added to reconstituted mononucleosomes. Binding was assayed by EMSA and visualized by ethidium bromide staining

As suggested by the structural analysis, recombinant UVR8 did not bind to reconstituted nucleosomes in vitro, either in its ground-state as homodimer or as UV-B-activated monomer (Fig. 5a and b). In contrast, recombinant RCC1 showed a clear electrophoretic mobility gel shift of the nucleosomes, demonstrating nucleosome binding (Fig. 5b).

## Conclusion

Contrary to the current models of UVR8 signalling, our data do not support binding of UVR8 to chromatin at target genes. Moreover, no chromatin-based activity of UVR8 could be demonstrated experimentally. However, it has been rather well established that UVR8 activity requires UV-B-dependent interaction with COP1, which leads to HY5 stabilization [7–9, 20]. HY5 associates with target gene promoters and is required for UV-B-induction of a large portion of the UV-B-induced genes [6, 10, 11, 27, 39]. Thus, we propose that the effect of UVR8-COP1 on gene expression is through HY5 and its homolog HYH, without involving direct UVR8 chromatin association at the same target genes.

However, we cannot exclude the existence under UV-B of short-lived and/or limited amounts of a possible UVR8-COP1-HY5 protein complex that was not detected by our qRT-PCR-based ChIP assays. Arguing against this is the evidence from previous gel-based ChIP assays which firstly suggested that UVR8 associates with the *HY5* promoter in the absence of UV-B and functional COP1 and secondly failed to detect chromatin association of GFP-COP1 [6, 7]. Moreover, no evidence was found for HY5 being part of a UVR8-COP1 complex [24], nor did we identify direct interaction of UVR8 with HY5 in yeast two-hybrid assays (Additional file 3). However, despite the prime role in UV-B signalling assigned to HY5, HY5 lacks a transcriptional activation domain and cannot alone activate transcription [27, 40]. Thus, to better understand how UVR8 regulates gene expression through HY5, we now need to identify HY5 partner protein(s) that provide a transcriptional activation domain and enable UV-B-responsive gene expression.

## Methods

### Plant material

*hy5-215* [41], *uvr8-6* [7], *Pro*_*35S*_*:CFP-UVR8* [1] and *hfr1-101* [42] are in the Arabidopsis Columbia (Col) accession; *Pro*_*HY5*_*:Luc +* [10], *uvr8-7* [7], *uvr8-7*/*Pro*_*HY5*_*:Luc +* [7], *uvr8-7*/*Pro*_*HY5*_*:Luc + Pro*_*35S*_*:UVR8* (“UVR8 OX”) [7], *hy5-ks50 hyh-1*/*Pro*_*HY5*_*:Luc +* [11] and *hy5-ks50* [41] are in the Wassilewskija (Ws) accession; *hy5-1* [41] and *hy5-1/Pro*_*HY5*_*:HY5-YFP* [43] are in the Landsberg *erecta* (L*er*) accession.

The *UVR8* full-length coding sequence was cloned into the Gateway-compatible vector pB7WGY2 [44]. The binary vector was transformed into *uvr8-7* to generate the *uvr8-7*/*Pro*_*35S*_*:YFP-UVR8* line. YFP-UVR8 is functional in this line as shown by a rescued hypocotyl growth inhibition phenotype under UV-B (Additional file 4).

### Growth conditions and light treatments

*Arabidopsis* seeds were surface-sterilized with sodium hypochlorite and plated on half-strength Murashige and Skoog medium (Duchefa) containing 1 % sucrose and 0.8 % agar. Seeds were stratified for at least 2 days at 4 °C and germinated aseptically at 25 °C in a standard growth chamber (MLR-350, Sanyo, Gunma, Japan) with 80 μmol m^−2^ s^−1^ white light and a 12-h/12-h light/dark cycle. UV-B irradiation was performed in a white-light field with Osram L18W/30 tubes (3.6 μmol m^−2^ s^−1^; measured with a LI-250 Light Meter) supplemented with Philips TL20W/01RS narrowband UV-B tubes (0.6 W m^−2^; measured with a VLX-3W Ultraviolet Light Meter equipped with a CX-312 sensor; Vilber Lourmat). The UV-B range was modulated by the use of 3-mm transmission cut-off filters of the WG series (Schott Glaswerke) with half-maximal transmission at the indicated wavelength (WG305 for + UV-B and WG360 for –UV-B conditions).

### ChIP assays

ChIP was performed as described before [11, 27]. The chromatin was immunoprecipitated with primary antibodies against HY5 [43], UVR8 (anti-UVR8^(410–424)^) [25] and GFP (A11122, Life Technologies). Quantitative real-time PCR ChIP data were obtained using the ABsolute SYBR Green Rox Mix Kit, according to the manufacturer’s instructions (Thermo Scientific). The samples were amplified using a 7900HT real-time PCR system (Applied Biosystems) with the primer pairs listed in Additional file 5. qPCR data were analysed according to the percentage of input method [45]. Technical error bars represent standard deviations of the means and were calculated according to the Applied Biosystems user manual.

### Protein gel blot analysis

Samples were taken during the ChIP procedure: (i) before adding the antibody for immunoprecipitation (“Input”) and (ii) after eluating the purified protein-chromatin-antibody complex from the beads (“IP: anti-GFP”). The samples were boiled for 10 min in SDS-PAGE buffer, separated by electrophoresis in 10 % SDS–polyacrylamide gels and electrophoretically transferred to PVDF membrane, according to the manufacturer’s instructions (Bio-Rad). We used monoclonal anti-GFP (Living Colors, 632381, Clontech) as primary antibody and horseradish-peroxidase-conjugated anti-mouse immunoglobulins (DAKO) as secondary antibody. Signal detection was performed using the Amersham ECL Select Western Blotting Detection Reagent (RPN 2235, GE Healthcare) and the Image Quant LAS 4000 mini CCD camera system (GE Healthcare).

### Transient protoplast assay

Gateway technology was used to clone *HY5* and *UVR8* coding sequences derived from Arabidopsis cDNA and a genomic fragment (from start ATG to stop codon) of *HFR1* [46] into the Gateway-compatible binary vectors pALLI1 [47] and pB2GW7 [44]. The resulting constructs, *Pro*_*35S*_*:3xHA-VP16AD-HY5, Pro*_*35S*_*:3xHA-VP16AD-UVR8, Pro*_*35S*_*:3xHA-VP16AD-HFR1, Pro*_*35S*_*:HY5*, *Pro*_*35S*_*:UVR8, Pro*_*35S*_*:HFR1* were verified by sequencing and corresponding Arabidopsis mutant protoplasts derived from *hy5-ks50, uvr8-7* and *hfr1-101* were transfected.

To test UV-B-responsiveness of protoplasts after transfection, protoplasts derived from *uvr8-7* and Ws were transfected with the HBT95:sGFP(S65T) vector [48] obtained from the ABRC (stock name: CD3-911). Protoplasts were isolated from 7-day-old seedlings grown in a standard growth chamber. Isolation and transfection procedures were adapted from a previously described protocol [49]. In brief, 10^5^ protoplasts were transfected with 25 μg vector DNA per experimental sample using the DNA-PEG-calcium transfection method. Protoplasts were incubated for 19–24 h under continuous white-light irradiation (3.6 μmol m^−2^ s^−1^). UV-B treated protoplasts were irradiated for 3 h in the white-light field supplemented with Philips TL20W/01RS narrowband UV-B tubes (0.6 W m^−2^; measured with a VLX-3W Ultraviolet Light Meter equipped with a CX-312 sensor; Vilber Lourmat) under a 3-mm transmission cut-off filter of the WG series with half-maximal transmission at 305 nm (Schott Glaswerke) [10]. UV-B treated and non-treated samples were harvested at the same time in liquid nitrogen. Gene expression was analysed by quantitative PCR. Expression was determined in technical triplicates. Gene expression levels were normalized with the expression levels of the transfected effectors. The effect of the VP16-fusion to effectors on target gene expression was assessed as relative expression in the presence of the effectors fused to VP16 to the respective control effectors without the VP16 domain (Fig. 3). Gene expression levels were normalized with the expression level of 18S and are presented relative to the expression level in Ws WT minus UV (Additional file 2). Data are shown as means and standard errors of four (Fig. 3a, b and c), three (Fig. 3d, e, and f)) or five (Additional file 2) biological replicates.

### Gene expression analysis

*Arabidopsis* protoplast RNA was isolated with the RNeasy kit (Qiagen) according to the manufacturer’s instructions. For quantitative real-time PCR analysis, total RNA was treated with DNaseI according to the manufacturer’s specifications (Qiagen). Per PCR reaction, cDNA was synthesized from 50 ng RNA with random hexamers using the TaqMan Reverse Transcription Reagents Kit (Applied Biosystems). Quantitative RT-PCR was carried out using a 7900HT real-time PCR system and TaqMan probes (Applied Biosystems). PCR reactions were performed using the ABsolute QPCR Rox Mix Kit following the manufacturer’s instructions (ABgene). The gene-specific probes and primers against *UVR8* (AT5G63860), *ELIP2* (AT4G14690), *MYB12* (At2G47460), *CHS* (At5G13930) and *HY5* (At5g11260) were used as previously described [7, 24, 27]. In the case of *HFR1*, the PCR reactions were performed using the ABsolute QPCR SYBR green Rox Mix Kit following the manufacturer’s instructions (ABgene) with HFR1-for (5’-GATGCGTAAGCTACAGCAACTCGT-3’) and HFR1-rev (5’-AGAACCGAAACCTTGTCCGTCTTG-3’), as previously described [50]. 18S rRNA transcript levels were analysed using the Eukaryotic 18S rRNA Kit (Applied Biosystems). Expression was determined in technical triplicates.

### Nucleosome reconstitution

Recombinant Xenopus core histones H2A, H2B, H3 and H4 were expressed in *E.coli* strain BL21pLysS, purified from inclusion bodies under denaturing conditions, refolded at equimolar ratios in 2M NaCl and reconstituted by salt dialysis with the MMTV DNA sequence into nucleosome core particles as described previously [51].

### Electrophoretic mobility gel shift assay

100nM of reconstituted mononucleosomes was mixed with varying concentration of recombinant RCC1 (ab90625, Abcam) and recombinant UVR8 (expressed and purified according to ref. [32]) from 10 μM to 75nM in buffers of 100 mM NaCl, 20 mM HEPES pH 7.6. The samples were incubated on ice for 1 h. Sucrose was then added to a final concentration of 5 % and the samples were electrophoretically separated in a 5 % native PAGE in 0.25xTBE buffer. For visualization the gel was stained with ethidium bromide.

### Yeast two-hybrid assay

Yeast two-hybrid experiments in L40 yeast cells with HY5 fused to the LexA DNA binding domain and COP1 fused to the GAL4 activation domain were performed as previously described [1, 9].

### Availability of supporting data

The data sets supporting the results of this article are included within the article and its additional files.

## Additional files

Additional file 1:
**HY5 does but UVR8 does not associate with the** ***HY5 genomic region.*** (PDF 134 kb) Additional file 2:
**UVR8 is functional as a UV-B photoreceptor in Arabidopsis protoplasts.** (PDF 72 kb) Additional file 3:
**UVR8 and HY5 do not interact in yeast.** (PDF 143 kb) Additional file 4:
**Overexpression of YFP-UVR8 restores UV-B photomorphogenesis.** (PDF 362 kb) Additional file 5:
**List of primers used for ChIP-qPCR in this work.** (PDF 104 kb)

## Abbreviations

CFP: cyan fluorescent protein
ChIP: chromatin immunoprecipitation
*CHS*: *CHALCONE SYNTHASE*
COP1: CONSTITUTIVELY PHOTOMORPHOGENIC 1
*CRY3*: *CRYPTOCHROME 3*
*ELIP2*: *EARLY LIGHT-INDUCIBLE PROTEIN 2*
GEF: guanine nucleotide exchange factor
GFP: green fluorescent protein
GTPase: guanosine triphosphate hydrolase
HFR1: LONG HYPOCOTYL IN FAR-RED 1
HY5: ELONGATED HYPOCOTYL 5
*HYH*: *HY5-HOMOLOG*
*Luc*: *LUCIFERASE*
*PFG1*/*MYB12*: *PRODUCTION OF FLAVONOL GLYCOSIDES 1/MYB DOMAIN PROTEIN 12*
*Pro*_*35S*_: cauliflower mosaic virus 35S promoter
*Pro*_*HY5*_: *HY5* promoter
qRT-PCR: quantitative reverse transcription polymerase chain reaction
Ran: Ras-related nuclear protein
RCC1: Regulator of Chromatin Condensation 1
Srm1/Prp20: Suppressor of receptor mutations 1/pre-RNA processing 20
UV-B: ultraviolet-B
UVR8: UV RESISTANCE LOCUS 8
VP16: Herpes Simplex Virus virion protein 16 acidic activation domain
WT: wild type
YFP: yellow fluorescent protein
