# Supplementary material for: Revisiting chromatin binding of the Arabidopsis UV-B photoreceptor UVR8
Source: BMC Plant Biol. 2016 Feb 11;16:42. doi: 10.1186/s12870-016-0732-5 (PMC4750278; doi:10.1186/s12870-016-0732-5)
Supplement: Additional file 1: — HY5 does but UVR8 does not associate with the HY5 genomic region. (PDF 134 kb) [file 12870_2016_732_MOESM1_ESM.pdf]

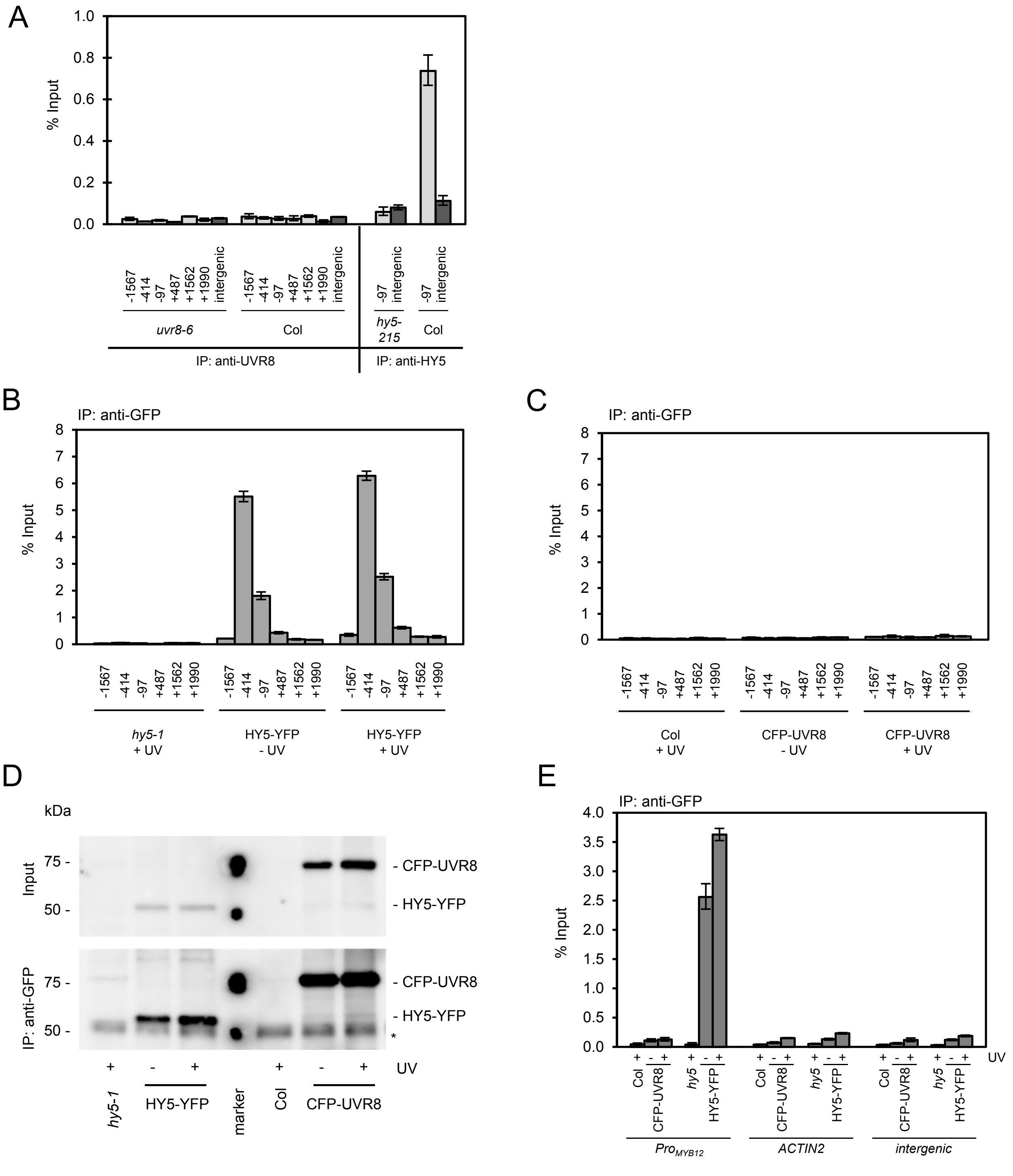


**Additional file 1.** HY5 does but UVR8 does not associate with the *HY5* genomic region. **a** UVR8 chromatin association in wild-type plants (Col) was compared to that in an *uvr8-6* mutant and HY5 chromatin association in wild-type plants (Col) was compared to that in a *hy5-215* mutant. ChIP-qPCR, presented as the percentage of total input DNA recovered by immunoprecipitation (% Input), was performed using an anti-UVR8$^{(410\text{-}424)}$ antibody (*left*) or an anti-HY5 antibody (*right*) for immunoprecipitation of UVR8 or HY5, respectively. Chromatin association was analysed by ChIP-qPCR using primer pairs covering the *HY5* genomic locus and surrounding region (see Fig. 1a) as well as an intergenic control region between the *At4g26900* and *At4g26910* genes to control sequence specificity. Experimental material was sourced from ten-day-old seedlings, either processed with no UV-B exposure (-UV) or following 4 h UV-B (+UV). **b, c** As for (**a**), but using the same anti-GFP antibody for the immunoprecipitation of both HY5-YFP (**b**) and CFP-UVR8 (**c**) and experimental material was sourced from seven-day-old seedlings. HY5-YFP = *hy5-1*/$Pro_{35S}$*:HY5-YFP*, CFP-UVR8 = $Pro_{35S}$*:CFP-UVR8*. The corresponding background *Arabidopsis* lines (*hy5-1* and wild-type Col) were included as negative controls. **a-c** The number of each analysed DNA fragment indicates the position of the 5' end of the amplicon relative to the *HY5* translation start site (referred to as position +1). Error bars represent SDs of three technical replicates. **d** Input and eluate of B/C ChIP samples were analysed on western blots with an antibody against GFP. Band resulting from unspecific background signal are marked (*). Molecular weight marker positions representing 50 and 75 kDa are shown (marker lane). **e** CFP-UVR8 does not display the same genomic association at the *MYB12* promoter as HY5. Chromatin association in HY5-YFP expressing plants (*hy5-1*/$Pro_{35S}$*:HY5-YFP*), a CFP-UVR8 line ($Pro_{35S}$*:CFP-UVR8*), and *hy5-1* and Col controls. ChIP-qPCR was performed using an anti-GFP antibody for immunoprecipitation. Chromatin association was analysed by ChIP-qPCR for the *MYB12* promoter ($Pro_{MYB12}$), *ACTIN2* and an intergenic control region between the *At4g26900* and *At4g26910* genes. The analysed chromatin was the same as in experiments presented in **b** and **c**. Error bars represent SDs of three technical replicates.
