# Supplementary material for: Revisiting chromatin binding of the Arabidopsis UV-B photoreceptor UVR8
Source: BMC Plant Biol. 2016 Feb 11;16:42. doi: 10.1186/s12870-016-0732-5 (PMC4750278; doi:10.1186/s12870-016-0732-5)
Supplement: Additional file 2: — UVR8 is functional as a UV-B photoreceptor in Arabidopsis protoplasts. (PDF 72 kb) [file 12870_2016_732_MOESM2_ESM.pdf]

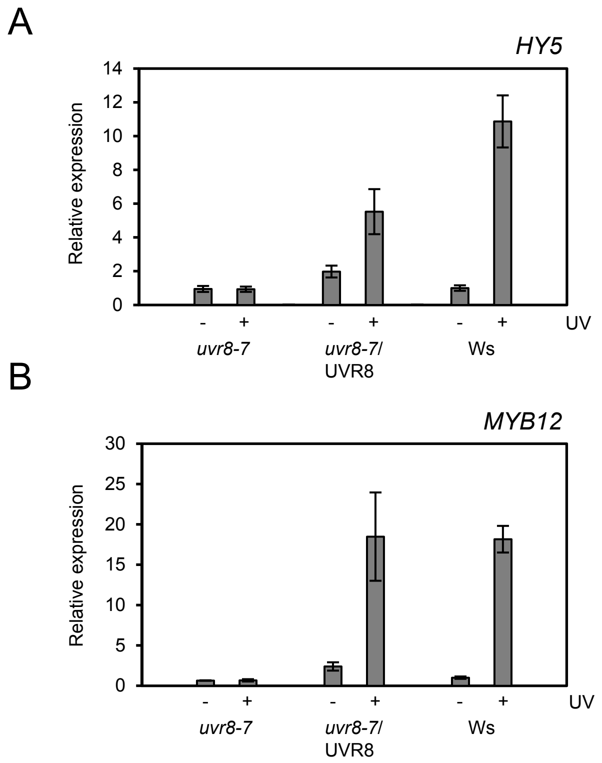

Additional file 2. UVR8 is functional as a UV-B photoreceptor in Arabidopsis protoplasts. **a, b** *uvr8-7* mutant and Ws wild-type protoplasts were transfected with sGFP(S65T) alongside *uvr8-7* mutant protoplasts transfected with Pro_{35S}*:UVR8* (*uvr8-7*/UVR8). Gene expression was analysed 19–24 h post-transfection with protoplasts maintained under weak white light (-) or weak white light plus a final 3 h UV-B irradiation (+). Relative gene expression of *HY5* (**a**) and *MYB12* (**b**) are shown; wild type (Ws) transfected protoplasts in white light without UV-B (i.e. - UV) were set = 1. Data are shown as the means of five biological replicates ± standard errors.
