# Supplementary material for: Revisiting chromatin binding of the Arabidopsis UV-B photoreceptor UVR8
Source: BMC Plant Biol. 2016 Feb 11;16:42. doi: 10.1186/s12870-016-0732-5 (PMC4750278; doi:10.1186/s12870-016-0732-5)
Supplement: Additional file 3: — UVR8 and HY5 do not interact in yeast. (PDF 143 kb) [file 12870_2016_732_MOESM3_ESM.pdf]

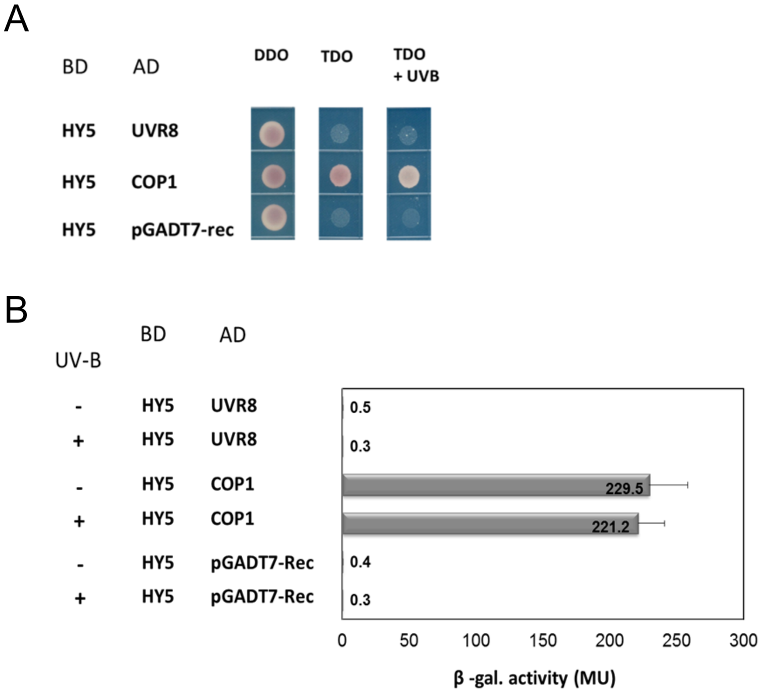

Additional file 3. UVR8 and HY5 do not interact in yeast. **a, b** Yeast two-hybrid interaction of UVR8 and HY5 in yeast growth assays (**a**) and quantitative lacZ assays (**b**) under white light (- UV-B) or supplemental UV-B (+ UV-B). HY5-COP1 interaction is shown as a positive control. Yeast growth medium DDO: SD/-Trp/-Leu; TDO: SD/-Trp/-Leu/-His. BD: LexA DNA-binding domain; AD: GAL4 activation domain.
