# Supplementary material for: Revisiting chromatin binding of the Arabidopsis UV-B photoreceptor UVR8
Source: BMC Plant Biol. 2016 Feb 11;16:42. doi: 10.1186/s12870-016-0732-5 (PMC4750278; doi:10.1186/s12870-016-0732-5)
Supplement: Additional file 4: — Overexpression of YFP-UVR8 restores UV-B photomorphogenesis. (PDF 362 kb) [file 12870_2016_732_MOESM4_ESM.pdf]

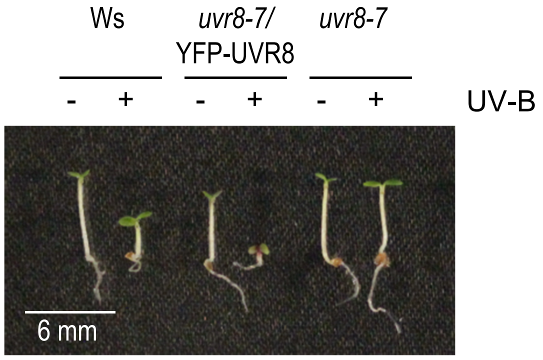

Additional file 4. Overexpression of YFP-UVR8 restores UV-B photomorphogenesis. *uvr8-7* seedlings expressing a Pro_{35S}:YFP-UVR8 construct in Ws background, wild type (Ws) and *uvr8-7* mutant seedlings were grown for 4 days under weak white light with or without supplementary UV-B. Images of representative seedlings are shown.
