# Supplementary material for: Revisiting chromatin binding of the Arabidopsis UV-B photoreceptor UVR8
Source: BMC Plant Biol. 2016 Feb 11;16:42. doi: 10.1186/s12870-016-0732-5 (PMC4750278; doi:10.1186/s12870-016-0732-5)
Supplement: Additional file 5: — List of primers used for ChIP-qPCR in this work. (PDF 104 kb) [file 12870_2016_732_MOESM5_ESM.pdf]

Additional file 5. Oligonucleotide sequences for the primer pairs used in ChIP-qPCR.

ChIP-qPCR

*	accession number	fw primer	rv primer
Pro$_{MYB12}$*_-302*	At2G47460	CGAGTCTCTCGACGGTGAATAA	GCGCCACGTTGGTTTAGAA
HY5_-1567	At5g11270	GATGACAGCGAAGAAGATGAAAGA	ACGCCCAGCTTTCAATGC
Pro$_{HY5}$*_-414*	At5g11260	GGCCATGTGACAGAATGAAAG	AGGATCCAAAGGCAATTGAG
HY5_-97	At5g11260	GCTCTTTTCCTCTTTATCCTTTTCAC	TGTTCCTGCATTTTTCTTACTCTTTG
HY5_+487	At5g11260	GAAAGAGAACAAGCGGCTGAA	CCCATCACGCAACCGTTATT
HY5_+1562	At5g11260	CGCCATGGATTTGTAAACGA	CCCTACCCTCTTTGTCCGATT
HY5_+1990	At5g11260	TTCTCCTTCTTTTACCACTTCCATCT	TGCGGAAGCTGGTAAAGACA
intergenic	At4g26900/At4g26910	TCTTATAGTTGATTTCCTTTTGTTGACAGT	GCTGAGAAAGTGAACATACGTTGCT
ACTIN2	At3g18780	TGCTGGACGTGACCTTACTG	TTCCATCTCCTGCTCGTAGTC

* Where included numbers indicate the position of the 5’ end of the amplicon relative to the translation start site (referred to as position +1).
